# Thymoquinone-Induced Tristetraprolin Inhibits Tumor Growth and Metastasis through Destabilization of MUC4 mRNA

**DOI:** 10.3390/ijms20112614

**Published:** 2019-05-28

**Authors:** Se-Ra Lee, Jeong-Yeon Mun, Mi-So Jeong, Hyun-Hee Lee, Yun-Gil Roh, Won-Tae Kim, Min-Hye Kim, Jeonghoon Heo, Yung Hyun Choi, Su Jin Kim, Hee-Jae Cha, Mira Jun, Sun-Hee Leem

**Affiliations:** 1 Department of Biological Science, Dong-A University, Busan 49315, Korea; srlee@kbiohealth.kr (S.-R.L.); moonmihye90@hanmail.net (J.-Y.M.); dkwl523@hanmail.net (M.-S.J.); hyunhee517@hotmail.com (H.-H.L.); royunkil@gmail.com (Y.-G.R.); kimwt33@nate.com (W.-T.K.); kmhmary93@naver.com (M.-H.K.); 2 Division of Drug Development & Optimization, Osong Medical Innovation Foundation (KBio), Chungbuk 28160, Korea; 3 Departments of Molecular Biology and Immunology, College of Medicine, Kosin University, Busan 49267, Korea; jeonghoonheo@kosin.ac.kr; 4 Department of Biochemistry, College of Oriental Medicine, Anti-Aging Research Center, Dongeui University, Busan 47227, Korea; choiyh@deu.ac.kr; 5 Department of Pathology, College of Medicine, Dong-A University, Busan 49315, Korea; tina_branf@naver.com; 6 Department of Parasitology and Genetics, Kosin University College of Medicine, Busan 602-702, Korea; hcha@kosin.ac.kr; 7 Department of Food Science and Nutrition, Dong-A University, Busan 49315, Korea; mjun@dau.ac.kr

**Keywords:** TTP, TQ, MUC4, ARE, binding protein, bioactive substance

## Abstract

Tristetraprolin (TTP), a well-characterized AU-rich element (ARE) binding protein, functions as a tumor suppressor gene. The purpose of this study was to investigate whether a bioactive substance derived from a natural medicinal plant affects the induction of TTP and to elucidate its mechanism. We examined the effects of natural bioactive materials including Resveratrol (RSV), thymoquinone (TQ) and curcumin on the expression of TTP in cancer cell. TQ derived from a natural plant *Nigella sativa* increased the expression levels of TTP mRNA and proteins in a dose-dependent manner in gastric and breast cancer cells. TQ-induced TTP increased the instability of MUC4 mRNA by direct binding of TTP to ARE in the 3′UTR of MUC4 mRNA. The induction of TTP by TQ also reduced the proliferation, migration and invasion of cancer cells. The expression of the epithelial-mesenchymal (EMT)-related genes, which were target genes of TTP, was also decreased by the TQ treatment. In the in vivo experiments using mouse melanoma cells, TQ-induced TTP inhibited metastasis of tumor cells. We have found that TQ-induced TTP might inhibit metastasis by reducing tumor cell migration and invasion through destabilization of MUC4 mRNA, which suggest the MUC4 as a novel target to TTP.

## 1. Introduction

Post-transcriptional regulation controls gene expression at the RNA level, which changes the state of the transcribed RNA prior to translation. Tristetraprolin (TTP) is known to be one of the RNA binding proteins (RBPs) that bind to the AU-rich element (ARE) at the 3‘UTR of the transcript; the target genes of TTP include inflammatory cytokines and proto-oncogenes [1,2,3,4]. TTP has also been reported to function as a tumor suppressor that inhibits the expression of many cell cycle progression and survival regulators [2,5,6,7]. Additionally, dysregulation of TTP has been reported to be associated with poor prognosis in several types of tumors [8,9,10]. Therefore, if TTP, known as a tumor suppressor gene, is induced as a biologically active substance, it may be useful as an adjunct to anticancer drugs.

It is known that natural bioactive substances have few side effects in the treatment of cancer patients and are of great importance because they increase the effectiveness of anticancer drugs in combination therapy [11,12]. Therefore, if cancer patients can be treated with natural physiologically active substances to induce the effect of cancer suppression, it is expected to have little side effects. Several studies on natural bioactive materials such as thymoquinone (TQ), Resveratrol (RSV) and curcumin have reported that these substances enhance susceptibility to chemotherapy [13,14,15]. TQ, a natural bioactive substance isolated from *Nigella sativa* oil, has been used in antioxidant and anti-inflammatory medicines for asthma, gastrointestinal disease and hypertension and growth inhibitory effects of TQ on several cancer cells [10,16], [17,18]. RSV (3,5,4′-trihydroxystilbene) is a naturally occurring polyphenolic compound in grapes, peanuts and berries [19]. In studies using a variety of human cancer types, the suppressive nature of RSV has also been reported [20,21,22].

In this study, we examined whether natural bioactive substances treatment induces TTP expression and how TTP acts as a tumor suppressor. As a result, we found that the natural bioactive TQ mediates the regulation of TTP expression. Furthermore, we have demonstrated that TQ induces ARE-containing mRNA degradation activity of TTP and that MUC4 is a novel target for the activity of these TTPs. Overall, we suggest that TQ induces transcription of TTP and destabilizes mRNA of MUC4 oncogene by this TTP activity, resulting in the inhibition of tumor growth and metastasis.

## 2. Results

### 2.1. TQ-Induced TTP Regulates MUC4 Expression in Cancer Cells 

**Figure 1:**
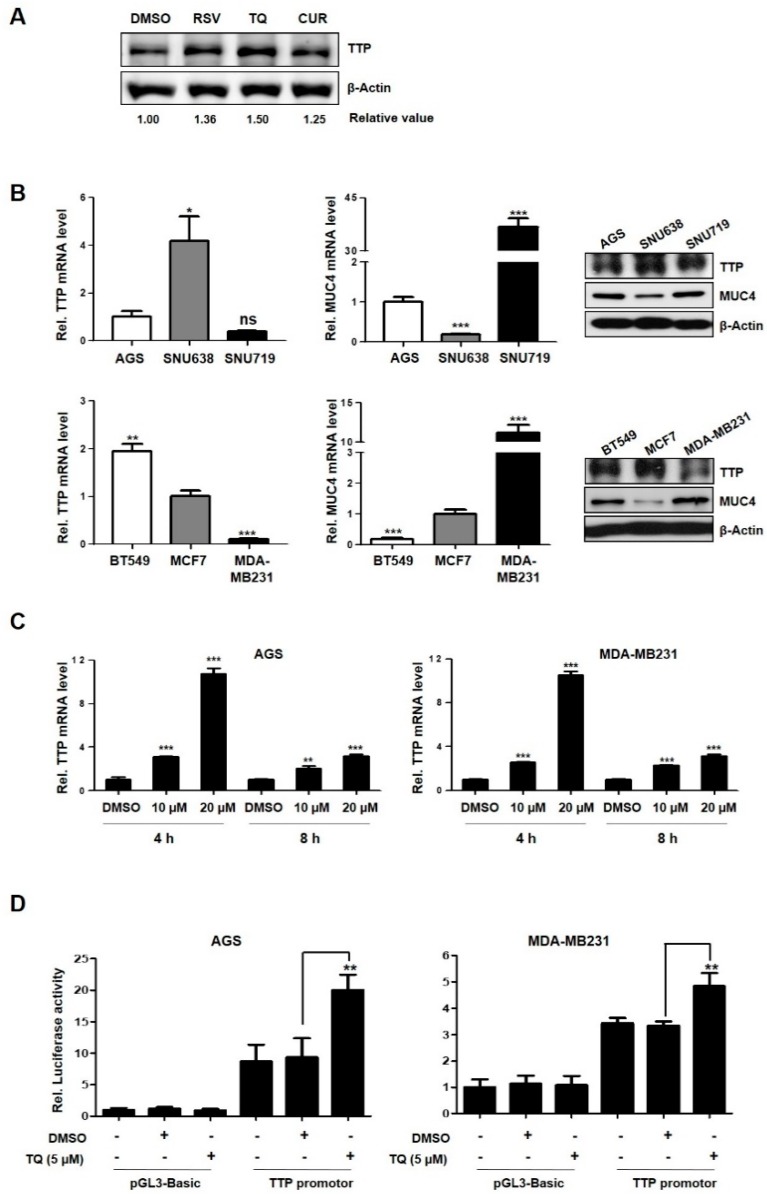
Thymoquinone (TQ) regulates MUC4 expression through the induction of tristetraprolin (TTP) expression. (**A**) AGS cells were treated with Resveratrol 20 µM, TQ 5 µM, curcumin 10 µM to induce TTP expression. TTP protein expression levels were confirmed by western blotting. The relative protein band density of TTP was normalized to β-Actin. (**B**) The endogenous level of TTP and MUC4 expression in gastric and breast cancer cell lines to qRT-PCR and western blotting. (**C**) AGS and MDA-MB231 cells were treated with TQ in a dose-dependent manner for 4 and 8 h. The mRNA level of TTP was determined by qRT-PCR as compared with DMSO as a control. β-actin was detected as a control for qRT-PCR. (**D**) AGS and MDA-MB231 cells were transfected with pGL3/TTPp-1411 containing the TTP promoter (−1343 to +68) for 24 h and then treated with TQ 5 µM. After treatment with TQ for 4 h, luciferase activity was measured. The expression levels obtained from pGL3 basic transfected cells without TQ treatment were set to 1. Each bar represents the mean ±S.D. of three independent experiments. Each bar represents the mean ± S.D. of three independent experiments. (* *p* < 0.05; ** *p* < 0.01; *** *p* < 0.001).

We investigated whether treatment of various natural bioactive materials using AGS cell lines could increase TTP expression. As a result, induction of TTP protein expression was observed in the RSV and TQ treatment, but not in the curcumin treatment (Figure 1A). Among these, the TQ treatment showed the most effective induction of TTP. One of the target genes for TQ had been reported in previous studies, MUC4, which is composed of a glycosylated alpha subunit and a beta subunit that is anchored to the cell membrane and extends into the cytosol [23]. This beta subunit acts as a ligand for ErbB2 and is considered an oncogene [24]. It has also been reported that TQ inhibits the expression of MUC4 and induces apoptosis in pancreatic cancer [25].

Based on previous study that MUC4 was decreased by TQ, we investigated the association of TQ with TTP and MUC4. First, the endogenous expression of TTP and MUC4 were examined in gastric and breast cancer cell lines using qRT-PCR and western blotting (Figure 1B). As shown in Figure 1B, the expression levels of TTP and MUC4 were variable in cancer cells. Two cell lines (AGS and MDA-MB231; gastric and breast cancer cells) with low levels of TTP expression and high levels of MUC4 expression were selected and used in subsequent experiments.

To investigate whether TQ induces TTP expression in cells, MUC4 and TTP expression levels were measured by qRT-PCR after treatment with various concentrations of TQ for 4 and 8 h. As observed in previous studies [25], the expression of TTP, a tumor suppressor gene, increased according to the concentration of TQ, and it was found that TTP was induced by TQ in a dose-dependent manner (Figure 1C, Supplementary Figure S1). Next, we used a luciferase reporter assay to test whether TQ treatment increased TTP promoter activity. Cells were transfected with a TTP promoter vector in which the TTP promoter region (−1343–+68) was inserted into a pGL3 basic vector [5], and then treated with TQ (5 μM) to investigate the promoter activity. The results show that the promoter activity of TTP was significantly increased in both cell lines treated with TQ (Figure 1D). 

**Figure 2:**
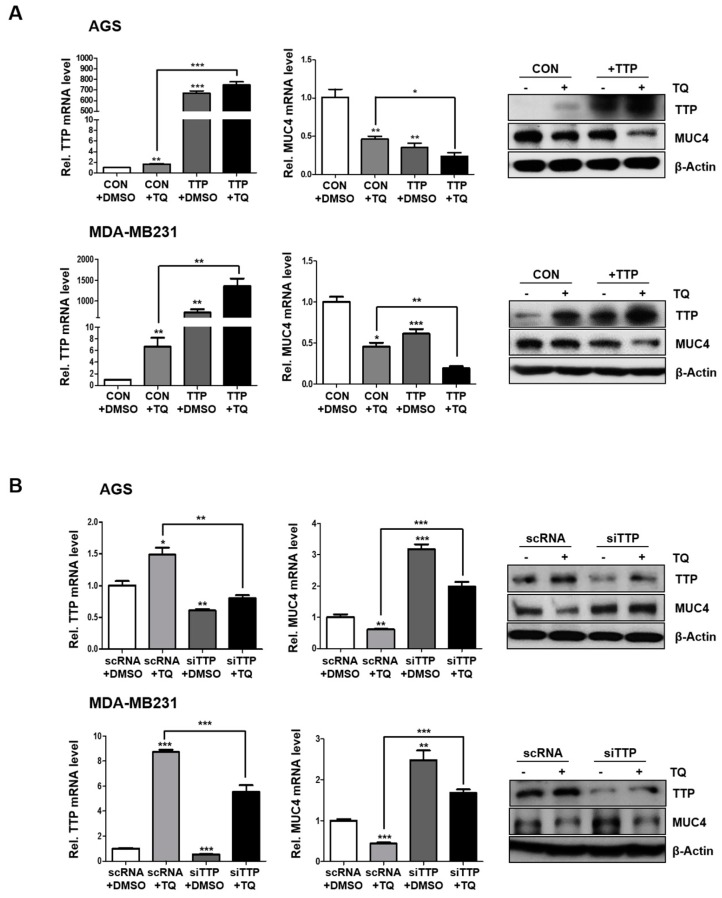
MUC4 expression is down-regulated by TTP expression in gastric and breast cancer cells. (**A**) Detection of MUC4 mRNA and protein in AGS and MDA-MB231 cells with transfected pcDNA6/V5 or pcDNA6/V5-TTP. (**B**) MUC4 mRNA and protein levels increased through inhibition of TTP. β-actin was detected as a loading control for qRT-PCR and western blotting. Each bar represents the mean ± S.D. of three independent experiments. * *p* < 0.05; ** *p* < 0.01; *** *p* < 0.001.

In order to investigate the correlation between TQ-mediated TTP and MUC4 genes, we investigated whether TTP affects MUC4 expression. We transiently transfected with the TTP-overexpressing vector (+TTP) and the negative control vector (CON) into cells (Figure 2A). Conversely, to investigate the effect of TTP depletion on MUC4 expression, cancer cells were transformed with scRNA and siTTP (Figure 2B). As the results show, expression of MUC4 mRNA and protein decreased when cells were transfected with TTP-overexpressing vector, whereas the expression of MUC4 increased with siTTP treatment in both cells (Figure 2A,B). Overall, the results show that the expression of MUC4 was down-regulated by TTP, suggesting the possibility of a new target for the post-transcriptional regulator TTP.

### 2.2. MUC4 mRNA was Identified as a Novel Target for TTP 

**Figure 3:**
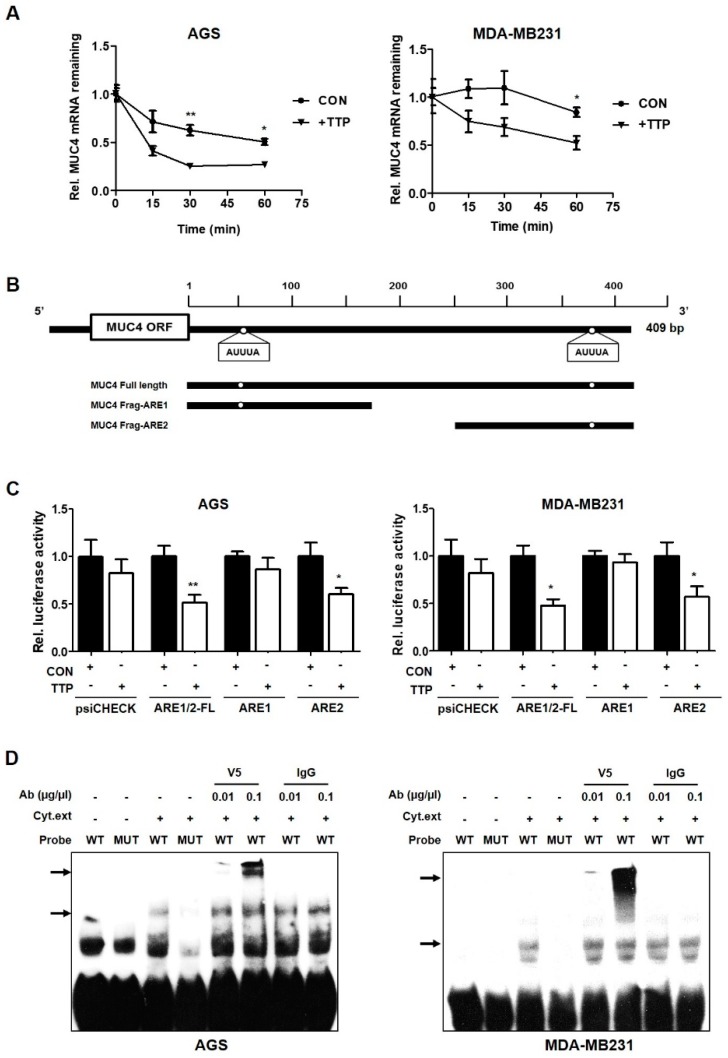
TTP binds directly to the ARE in the 3’UTR of MUC4 mRNA and makes it unstable. (**A**) After addition of actinomycin D to cells transfected with pcDNA6/V5 or pcDNA6/V5-TTP, the remaining MUC4 mRNA was examined. * *p* < 0.05; ** *p* < 0.01. (**B**) A schematic map of the luciferase reporter constructs. The two white circles represent the binding motif AUUUA in the MUC4 3′UTR. The ARE1 construct contains first AUUUA and ARE2 involved a second AUUUA. (**C**) Cells were co-transfected with a luciferase reporter vector (containing three types of AREs in the MUC4 3’UTR region) and a pcDNA6/V5-TTP or empty pcDNA6/V5 control vector. Renilla luciferase activity was normalized to firefly activity. The luciferase values obtained from cells co-transfected with a luciferase construct and pcDNA6/V5 were set to 1. The results shown on the graph represent the mean ± S.D. of three independent experiments. * *p* < 0.05; ** *p* < 0.01. (**D**) An RNA-EMSA assay was performed by mixing cytoplasmic extracts containing 10 μg of total protein with 10 fmol of biotinylated wild-type (WT) probe (AUUUA) and a mutant probe (AGCGA). Anti-TTP and control antibodies were added to the reaction mixtures. The binding reactions were then separated by electrophoresis on a 5% polyacrylamide gel under non-denaturing conditions. Arrows indicate the position of the TTP-containing band.

We investigated whether TTP affects MUC4 mRNA stability by measuring the half-life of this mRNA in cells transfected with TTP-overexpressing or control vector. Using actinomycin D as a transcriptional inhibitor, we confirmed that amount of MUC4 mRNA by qRT-PCR. While MUC4 mRNA was stable until 30 min after actinomycin D treatment in control vector-transfected cells, the half-life was significantly reduced at 30 min after treatment in TTP-transfected cells (Figure 3A). This result indicates that elevated TTP expression contributes to a decrease in MUC4 levels through the destabilization of MUC4 mRNA.

Based on the results that TTP is associated with the instability of MUC4 mRNA, we analyzed the 3’UTR of MUC4 and identified two AU-rich elements (ARE1 and ARE2; Figure 3B). To confirm whether these two AREs are responsible for TTP activity, we constructed plasmids that inserted WT-ARE1+ARE2(ARE1/2-FL), WT-ARE1 or WT-ARE2 into a psiCHECK luciferase reporter vector (Figure 3B,C). Luciferase activity was significantly reduced when transfected with a vector containing the ARE1/2-FL or ARE2 region, but no effect was observed using the vector containing the ARE1 (Figure 3C). This result indicates that ARE2 is an important region for the destabilization of MUC4 mRNA by TTP.

Next, we confirmed by RNA-Electrophoretic Mobility Shift(EMSA) assay that TTP directly binds to ARE2 in the mRNA 3’UTR of MUC4 (Figure 3D). The RNA probe for RNA EMSA was performed using a biotinylated RNA probe containing the wild-type (WT) or mutant (MUT) ARE2 of MUC4 as the same region of the ARE2 fragment for luciferase analysis (Figure 3B,D). Cytoplasmic extracts were prepared from cells transfected with pcDNA6/V5-TTP and were incubated with the biotinylated RNA probe containing the wild-type or mutant ARE2 of MUC4. RNA-protein complexes were present in the cytoplasmic extracts of TTP-overexpressing cells with the WT-ARE2 sequence, but not when the mixture included MUT-ARE2 (Figure 3B,D). In addition, when the mixture of cytoplasmic extracts was pre-incubated with a V5 antibody, a dose-dependent supershift band appeared, but no reaction was observed when the mixture was pre-incubated with anti-IgG as the negative control (Figure 3D). These results demonstrate that TTP makes MUC4 mRNA unstable by binding directly to ARE2 in the 3’UTR of MUC4.

### 2.3. TQ-Induced TTP Expression Inhibits Tumor Progression

**Figure 4:**
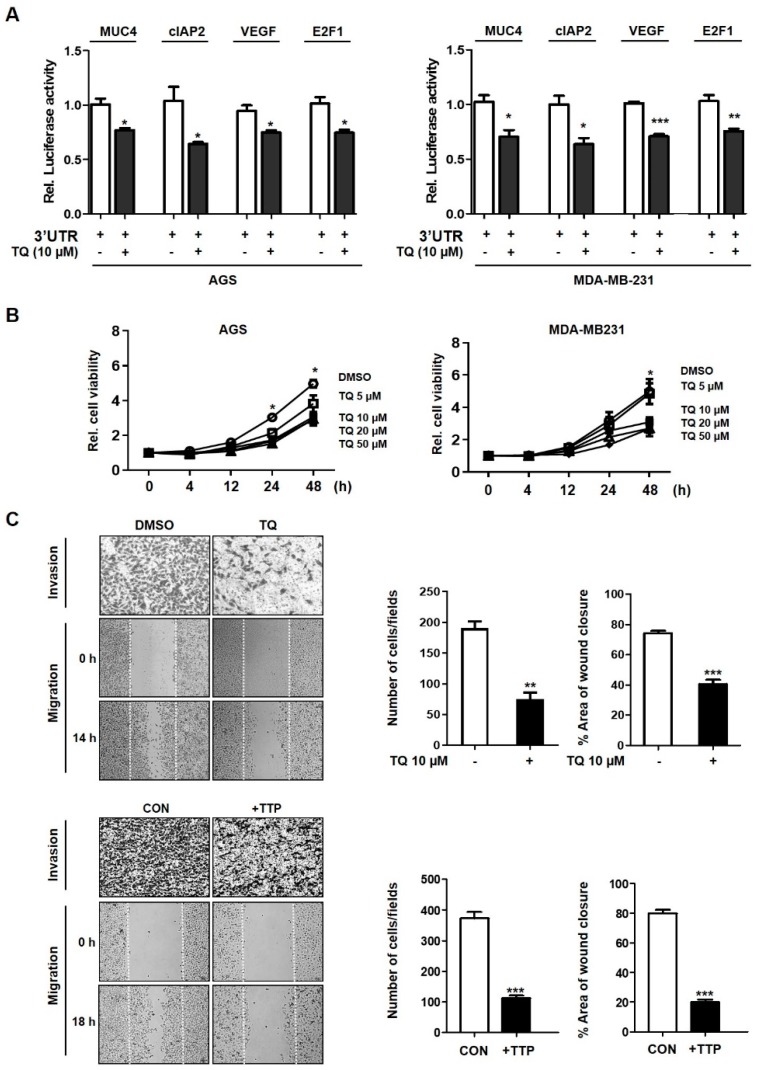
Induction of TTP expression by TQ inhibits tumor progression. (**A**) Cells were transfected with luciferase reporter constructs, psiCHECK2/MUC4, cIPA2, VEGF and E2F1 3′UTR mRNA after treatment with TQ. The luciferase activity obtained from psiCHECK2/MUC4, cIAP2, VEGF and E2F1 3′UTR transfected cells was set to 1. Each bar represents the mean ± S.D. of three independent experiments. * *p* < 0.05; ** *p* < 0.01; *** *p* < 0.001. (**B**) The viability of cancer cells was measured after TQ in the MTT assays. Cells treated with TQ in a dose-dependent manner were seeded at 1 × 10^4^ cells per well in 96-well plates. The data represent the mean ± S.D. of three different experiments. *p < 0.05. (**C**) AGS cells treated with TQ (upper panels) and transfected TTP overexpression vector (lower panels) were examined for the rate of invasive and migratory cells. Invading cells were visualized by staining the membranes using a Diff Quick solution, and the number of cells (five fields/well) was then counted. Migrating cells was measured interval that scratched after 14 h (TQ treatment) or 18 h (TTP overexpression). The graph showed the relative percentage area of wound closure (*** *p* < 0.001).

To investigate whether TQ treatment affects the destabilization of TTP target genes, the 3′-UTR luciferase reporter vectors containing the cIAP2, Vascular endothelial growth factor(VEGF) and E2F1 reported as TTP targets [26,27] and the newly identified MUC4 were transfected into cells. TQ treatment resulted in a decrease of luciferase activity and mRNA levels in cells containing the 3’UTR of the target gene of TTP (Figure 4A, Supplementary Figure S2A). To determine how TQ affects the progression of cancer, we examined the effects of proliferation, migration and invasion of cancer cells after treatment. TQ-treated cells were measured by the MTT assay and showed a dose-dependent inhibition of cell proliferation (Figure 4B). We also examined whether TQ treatment had the same effect as TTP overexpression in cancer cells on the migration and infiltration (Figure 4C). Cells treated with TQ were reduced in both cell migration and cell invasion compared to the controls (Figure 4C, upper panel). Similar results were also seen in cells transfected with the TTP overexpressing (Figure 4C, lower panel). Thus, TQ-induced TTP expression reduced the expression of the new target gene, MUC4, suggesting that such a decrease in MUC4 may inhibit cancer cell progression.

**Figure 5:**
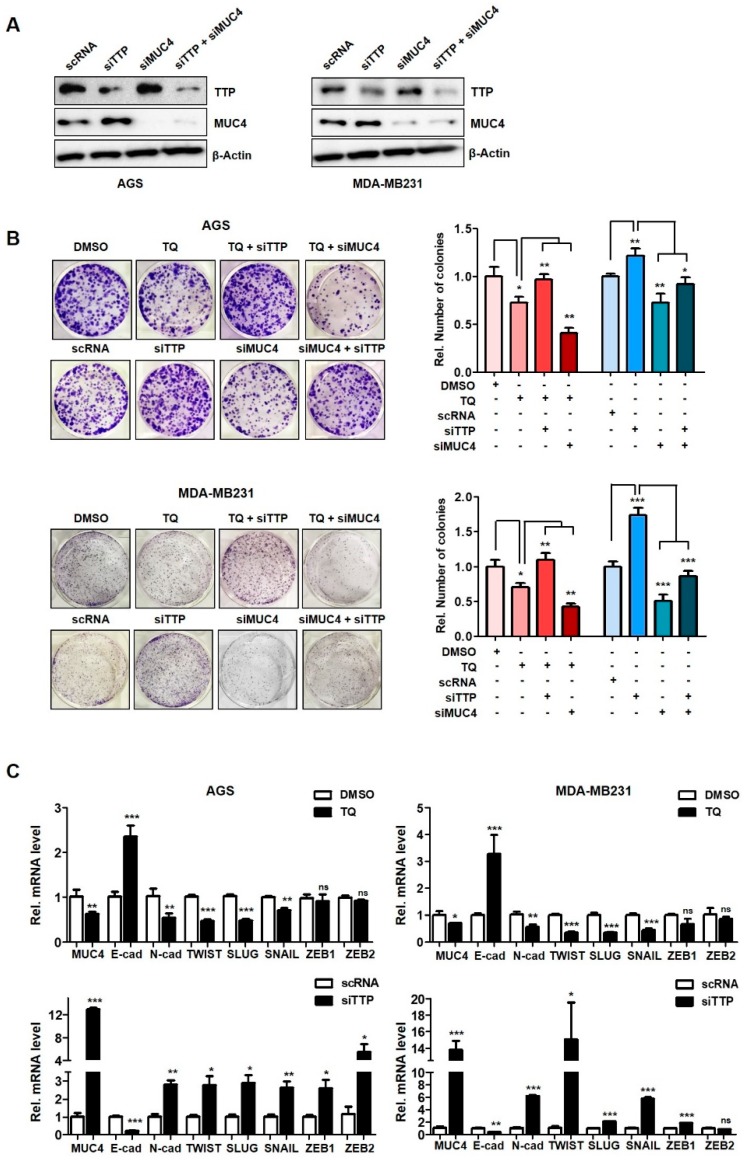
Suppression of TTP promotes proliferation and mesenchymal-related genes in cancer cells. (**A**) Western blotting was performed in AGS, MDA-MB-231 cells transfected with siTTP or/and siMUC4. (**B**) Clonogenic assay was performed in cells treated with TQ after transfection with siTTP or siMUC4 to assess proliferation. Cells were stained 0.05% crystal violet after fixation. (**C**) Analysis of mRNA expression of epithelial-mesenchymal (EMT)-related genes in two cell lines treated with TQ or transfected with siTTP. Several primer sets were used, such as E-cadherin, N-cadherin, TWIST, SLUG, SNAIL, ZEB and ZEB2, to determine MUC4 target genes. β-actin was used as a loading control for qRT-PCR and western blotting. Each bar represents the mean ± S.D. of three independent experiments (* *p* < 0.05; ** *p* < 0.01; *** *p* < 0.001).

To further confirm these results, conversely, the effect on the suppression of TTP and MUC4 was confirmed (Figure 5). The effects of siTTP and/or siMUC4 treatment on the expression of both genes were confirmed by western blotting (Figure 5A). TQ treatment reduced colony formation, but conversely siTTP treatment increased colony formation (Figure 5B). Decreased colony formation by TQ treatment was restored by treatment with siTTP at the same time (Figure 5B). That is, the increase in TTP due to TQ treatment was offset by simultaneous siTTP treatment. Transfection of siMUC4 simultaneously with TQ treatment further reduced colony formation compared to single treatment. However, co-transfection with siMUC4 and siTTP resulted in the elimination of both effects as described above (Figure 5B).

Expressions of N-cadherin, TWIST, SLUG, SNAIL, ZEB1 and ZEB2, which are EMT (epithelial-mesenchymal) markers regulated by MUC4, were also investigated after TQ treatment. As a result, E-cadherin mRNA expression was significantly increased in TQ-treated cells, in contrast to reduced MUC4 and mesenchymal markers (Figure 5C). Thus, TQ-induced TTP overexpression reduces target MUC4 expression and suggests that this reduction of the cancer gene, MUC4, may inhibit cancer cell proliferation and metastasis.

### 2.4. TQ-Induced TTP Expression Suppresses Metastasis In Vivo

**Figure 6:**
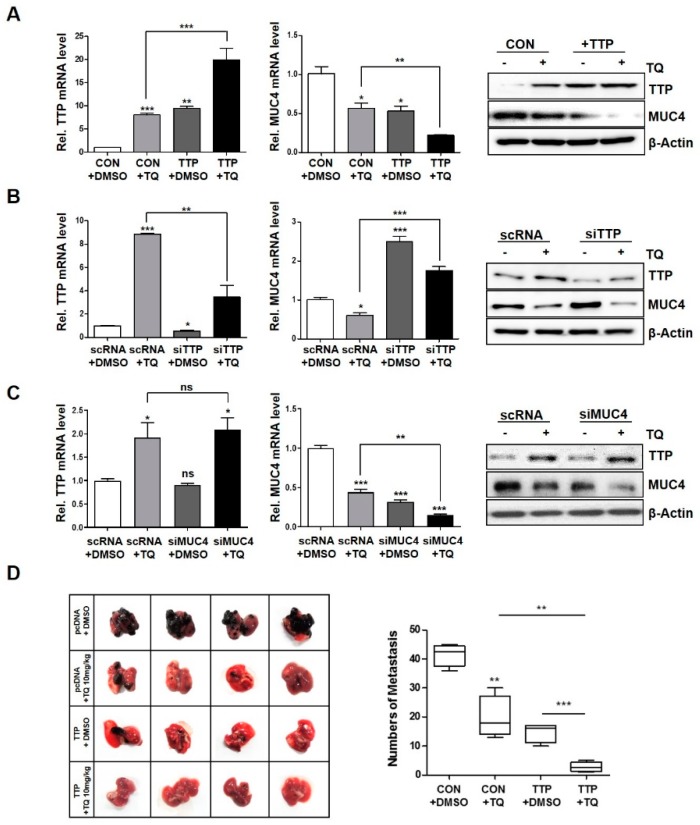
TQ-induced TTP reduced cancer metastasis in vivo. (**A**) TQ treatment and TTP overexpression reduce MUC4 expression in B16F10 cells. Cells were transfected with pcDNA (control; CON) or TTP (+TTP overexpression vector), exposed to TQ, and the expression of TTP and MUC4 was examined in these cells. After 24 h, mRNA and protein levels were analyzed using qRT-PCR (left panel) and western blotting (right panel). (**B**) Suppression of TTP by siTTP increased mRNA and protein levels of MUC4 in B16F10 cells. Cells were transfected with scRNA or siTTP and then exposed to TQ, and expression levels were analyzed by qRT-PCR and western blotting. (**C**) Cells were transfected with scRNA or siMUC4 and then treated TQ, and expression levels were analyzed by qRT-PCR and western blotting. β-actin was used as a loading control for qRT-PCR and western blotting. Each bar represents the mean ± S.D. of three independent experiments (ns: not significant; * *p* < 0.05; ** *p* < 0.01; *** *p* < 0.001). (**D**) B16F10 cells transfected with control or TTP overexpression vector were injected into mice tail vein. After 24 h, DMSO or TQ (10 mg/kg) was added to the drinking water. Black dots indicate metastasis in the mouse lungs (** *p* < 0.01; *** *p* < 0.001).

In the murine colorectal cancer model, treatment of TQ has been reported to reduce tumor invasion and inhibit cell growth [28]. We investigated the effect of TQ treatment and TTP overexpression on metastasis using mouse B16F10 cells. Similar to the results of using the previous human cancer cells, TTP expression was also induced in a TQ-dose dependent manner in cells (Supplementary Figure S3A). An MTT assay was performed in cells transfected by TTP overexpression vector. Cells were transfected with pcDNA (control) or TTP (pTTP overexpression vector), and the expression of TTP was examined in these cells (Supplementary Figure S3B). In addition to the increase in expression of TTP by the TQ treatment, the expression of MUC4 was also decreased by TTP overexpression and the TQ treatment in cells (Figure 6A). Conversely, in order to confirm the increase of MUC4 expression by TTP suppression, siTTP was treated in cells and the increase of mRNA and protein level of MUC4 was confirmed (Figure 6B).

To examine the effect of TTP overexpression and TQ treatment on cancer metastasis, cells transfected with control and TTP overexpression vectors were used to inject into the tail vein of C57BL/6 mice (Figure 6C). After injection, TQ group were fed TQ (10 mg/kg) in water for about one day and the control group were fed with an equivalent amount of DMSO. Three weeks later, mice were sacrificed and black spots were counted in the lungs to investigate metastasis (Figure 6C). TTP overexpression or TQ treatment significantly reduced cancer metastasis, and there was a synergistic effect when both treatments were simultaneously administered (Figure 6C). In previous studies using cancer cells, several studies have been reported on the anti-inflammatory and growth inhibitory effects of TQ [17,18]. In this in vivo experiment, we have shown that TQ induces TTP expression and reduces metastasis.

## 3. Discussion

TTP involved in the post-transcriptional regulation of gene expression, and this regulatory mechanism can lead to instability of many cytokines and cancer gene mRNAs [1,2,8]. Recent studies have shown that TTP is expressed at low levels in cancer tissues; it regulates oncogene-related cell growth, proliferation and metastasis, acting as a tumor suppressor [7,17,27,29]. We have previously investigated whether high expression of TTP correlates with decreased expression of oncogenes in various cancer cells [27,30]. Therefore, it is interesting to find natural bioactive substances that induce the expression of TTP.

TQ has been used in antioxidant and anti-inflammatory medicines in gastric, colon and pancreatic cancer cells in recent studies [17,25,31]. Additionally, TQ has been shown to suppress the proliferation and progression of cancer cells in several studies [16,17,18]. It has also been reported that TQ inhibits MUC4 expression, a primary oncogene associated with cell proliferation and progression of pancreatic cancer cells [25]. MUC4 is a membrane-bound mucin protein that plays a role in migration and invasion of cancer cells, and serves as a sensor for a variety of downstream cell signaling pathways [32,33,34].

In this study, we determined that TQ induced TTP expression at the mRNA and protein levels in gastric and breast cancer cells (Figure 1 and Figure 2). TQ-induced TTP inhibited the stability of MUC4 mRNA, confirming that MUC4 is a novel target gene for TTP. In addition, it was also confirmed that TTP directly binds to the ARE site in the 3’UTR of MUC4 (Figure 3). These results confirmed the MUC4 as a new target of TTP. We also observed a decrease in cancer invasion and migration from TQ treatment as well as a reduction in the expression of EMT-related genes, such as N-cadherin, TWIST, SLUG, SNAIL, ZEB1 and ZEB2, including downstream MUC4 (Figure 4 and Figure 5). TQ treatment also induced expression of TTP and decreased MUC4 expression in the B16F10 mouse cell line. In vivo experiments using these mouse cells confirmed a marked reduction in metastasis by TTP overexpression and TQ treatment (Figure 6). These results indicate that MUC4 mRNA is destabilized by TQ-induced TTP, resulting in inhibition of cancer proliferation and metastasis. In addition, we investigated the association with p38 in order to elucidate the mechanism of induction of TTP by TQ. The protein levels of c-RAF, P-MEK1/2, P-ERK, P-p38 and TTP were increased by TQ treatment (Supplementary Figure S4). These results suggest that TQ may result in destabilization of MUC4 mRNA by TTP via the Raf–MEK–ERK pathway. However, this result only provides limited information, so further studies are needed to elucidate a clear mechanism of TTP induction by TQ.

In recent years, there has been increased concern about the side effects of chemotherapy and radiation therapy on cancer and the recurrence of cancer after such treatments [35,36]. Many researchers are interested in natural bioactive substances for the treatment of relatively low-risk cancer as these compounds are expected to increase the efficacy of drugs in parallel with anticancer drugs in cancer patients [13,14,15]. We have been exploring the direct molecular mechanisms of natural biologically active materials such as TQ, which have been used for a long time but are known to be efficacious without revealing the exact mechanisms. This study has shown the potential of TQ, a bioactive substance that appears to play a direct role in cancer treatment, and provides insight into the pathways involved in cancer treatment with TQ. Our results would be a good example for appropriate application in the bio-food sector, such as the development of herbal medicines, as this approach shows the potential for drug treatment with fewer side effects in cancer patients.

## 4. Materials and Methods

### 4.1. Cell Lines and Reagents

Human gastric cancer cell lines (AGS, SNU638 and SNU719) and breast cancer cell lines (BT549, MCF7 and MDA-MB231), mouse melanoma cell line B16F10 were purchased from the Korean Cell Line Bank (KCLB). Human cancer cell lines were cultured in RPMI 1640 medium supplemented with 10% FBS and 1% penicillin/streptomycin (Capricorn scientific) at 37 °C in a humidified atmosphere of 5% CO_2_, and B16F10 was cultured in Dulbecco’s Modified Eagle Medium (DMEM) medium under the same conditions. TQ was suspended in dimethyl sulfoxide (DMSO) (Sigma-Aldrich, St. Louis, MO, USA). 

### 4.2. Small Interference RNA (siRNA)

For inhibition of TTP, MUC4 expression in human and mouse cancer cell lines, siRNAs were purchased from Santa Cruz (Human TTP siRNA, Mouse TTP siRNA, Human MUC4 siRNA, Santa Cruz biotechnology, TX, USA; Negative control RNA: synthesized Gene pharma, Shanghai, China). Each siRNA was treated with 100 nmol/mL into six wells for 24 h.

### 4.3. MTT and Clonogenic Assay

AGS, MDA-MB-231 cells were seeded at 1 × 10^4^ cells/well in 96-well culture plates and incubated with different concentrations of TQ (5, 10, 20 or 50 μM) for up to 48 h. In mouse B16F10 cells also transfected with pcDNA or TTP overexpression vector were seeded in 96-well culture plates and incubated for up to 72 h. MTT reagent (Thiazolyl Blue Tetrazolium Bromide) (Sigma-Aldrich) was added to each well according to the manufacturer’s instructions at the indicated times and the absorbance at 490 nm (OD490) was measured using a Wallac Victor3 1420 multilabel counter (Perkin Elmer, MA, USA).

AGS, MDA-MB231 cells were seeded at 1000 cells in 12-well culture plates and then transfected siTTP or/and siMUC4. TQ treated, cells were fixed and stained 0.05% crystal violet solution. After washing three times with PBS, pictures were taken and the number of stained colonies counted. 

### 4.4. Plasmid Construction and Luciferase Assay

The pcDNA5/V5/TTP overexpression construct, the pGL3/TTPp-1411 promoter construct, the psiCHECK2/E2F1 3′UTR, the psiCHECK2/VEGF 3′UTR and the psiCHECK2/cIAP2 3′UTR luciferase reporter construct have been described in our previous studies [16,17]. Two oligonucleotides containing ATTTA motifs of the MUC4 mRNA 3′UTR were synthesized at Integrated DNA Technologies (IDT, Coralville, IA, USA). The oligonucleotide sequences were as follows: Oligo-ARE1-WT (TCGAGAGGGGCAGCTGTG GCCTAGGCTACCTCAAGACTCACCTCATCCTTACCGCACAGCGAAGGCGCCATTG CTTTTGGGAGACTGGAAAAGGGAAGGTGACTGAAGGCTGTCAGGGC, GGCCGCTT GGAGACACAAAAAGTCAGAGAGACTTTATTTCGCTAGAGTTAATTTGAAGTAAACCAGAGAGTTTTGTGTGCAGAAGCATTTTGCTTAACTTAGGGCCATCACCACATTC); and Oligo-ARE2-WT (TCGAGAATGTGGTGATGGCCCTAAGTTAAGCAAAATGCTT CTGCACACAAAACTCTCTGGTTTACTTCAAATTAACTCTAGCGAAATAAAGTCTCTCTGACTTTTTGTGTCTCCAAGC, GGCCGCCCTGACAGCCTTCAGTCACCTTCCCTTT TCCAGTCTCCCAAAAGCAATGGCGCCTTCGCTGTGCGGTAAGGATGAGGTGAGTCTTGAGGTAGCCTAGGCCACAGCTGCCCCTC). Mutant oligonucleotides of ARE1 and ARE2, in which the ATTTA binding site sequences were substituted with AGCGA, were also synthesized. The oligonucleotides were ligated into the *Xho*I/*Not*I site of the psi-CHECK2 Renilla/firefly dual-luciferase expression vector (Promega, Madison, WI, USA). Cells were transfected with various kinds of plasmid constructs using iN-fect^TM^ in vitro transfection reagent (iNtRON, Burlington, MA, USA). For luciferase assays, AGS and MDA-MB231 cells were co-transfected with various types of psiCHECK2/cIAP2, E2F1, and VEGF 3′UTR constructs and a pGL3/TTPp-1411 promoter construct using iN-fect^TM^. Transfected cells were lysed with a lysis buffer and mixed with luciferase assay reagent, and the chemiluminescent signal was measured using a Wallace Victor3 1420 multilabel counter. Firefly luciferase was normalized to Renilla luciferase in each sample.

### 4.5. Western Blot Analysis

Western blotting was performed as described in our previous study [27]. Proteins were probed with the appropriate dilution of the following antibodies, anti-TTP (Sigma-Aldrich), anti-MUC4 (Abcam, Cambridge, UK), and anti-β-actin (Cell signaling, Danvers, MA, USA).

### 4.6. Quantitative Real-Time (qRT) PCR

qRT-PCR was performed using SYBR Premix Ex Taq (Takara, Japan) as described in our previous study [27]. The sequences of the qPCR primer pairs are listed in Supplementary Table S1.

### 4.7. Wound Healing and Invasion Assay

Cells were seeded in 12-well plates to 90% confluence. A wound was induced by scratching the cell cultures with a pipette tip. Following rinsing with a phosphate-buffered saline (PBS) to remove the detached cells, TQ were added to each well and incubated in a 5% CO_2_ incubator for 24 h at 37 °C. Images were immediately captured from each well and again after 14 or 18 h. The width of the wound at these specific locations was visualized on each plate to quantify the rate of cell migration. The invasion assay was performed as described in our previous study [27].

### 4.8. RNA Electrophoretic Mobility Shift Assay (RNA-EMSA)

The biotinylated RNA probes for wild-type (Oligo-MUC4-ARE2-WT, 5′-UACUUCAA AUUAACUCUAUUUAAAUAAAGUCUCUCUGAC-3′) and mutant (Oligo-MUC4-ARE2 -MUT, 5′-UACUUCAAAUUAACUCUAGCGAAAUAAAGUCUCUCUGAC-3′) were synthesized by Samchully Pharm. Co., Ltd. (Seoul, Korea). Cytoplasmic extracts were prepared from AGS and MDA-MB231 cells using NE-PER^®^ Nuclear and Cytoplasmic Extraction Reagent (Thermo Scientific, Rockford, IL, USA). RNA EMSA was performed as described in our previous study. For the supershift EMSA, anti-V5 antibody (Bethyl Laboratories, Montgomery, TX, USA) or control antibody was used.

### 4.9. Mouse In Vivo Experiment

Wild-type female C57BL/6 mice, six weeks of age, were injected with 1 × 10^6^ B16F10 melanoma cells transfected with control (pcDNA) and TTP overexpressing vector (+TTP) in the tail intravenously to induce melanoma. Mice were divided into four groups of four after melanoma cell inoculation and being treated with 0.1 mL of DMSO or 10 mg/kg body weight TQ dissolved in DMSO each day for three weeks by gavage. The body weight of each mouse was recorded every four days to determine whether the treatment influenced the animal’s health status. Twenty-one days after injection of the tumor cells, mice were sacrificed and explored when they appeared pre-morbid. The lungs were removed, and two independent observers determined the number of metastatic nodules on the lungs of each mouse.

### 4.10. Statistical Analysis

GraphPad Prism 5.0 (Graph Pad Software, San Diego, CA, USA) was used for all statistical analyses. Data are presented as means ± standard deviation (SD). For statistical comparisons, *p* values were determined using a Student’s t-test.

## Supplementary Materials

Supplementary Figures S1–S4 and supplementary Table S1 can be found at https://www.mdpi.com/1422-0067/20/11/2614/s1.
