# Supplementary material for: Thymoquinone-Induced Tristetraprolin Inhibits Tumor Growth and Metastasis through Destabilization of MUC4 mRNA
Source: Int J Mol Sci. 2019 May 28;20(11):2614. doi: 10.3390/ijms20112614 (PMC6600862; doi:10.3390/ijms20112614)
Supplement: Supplementary file 1 [file ijms-20-02614-s001.pdf]

Supplementary Material

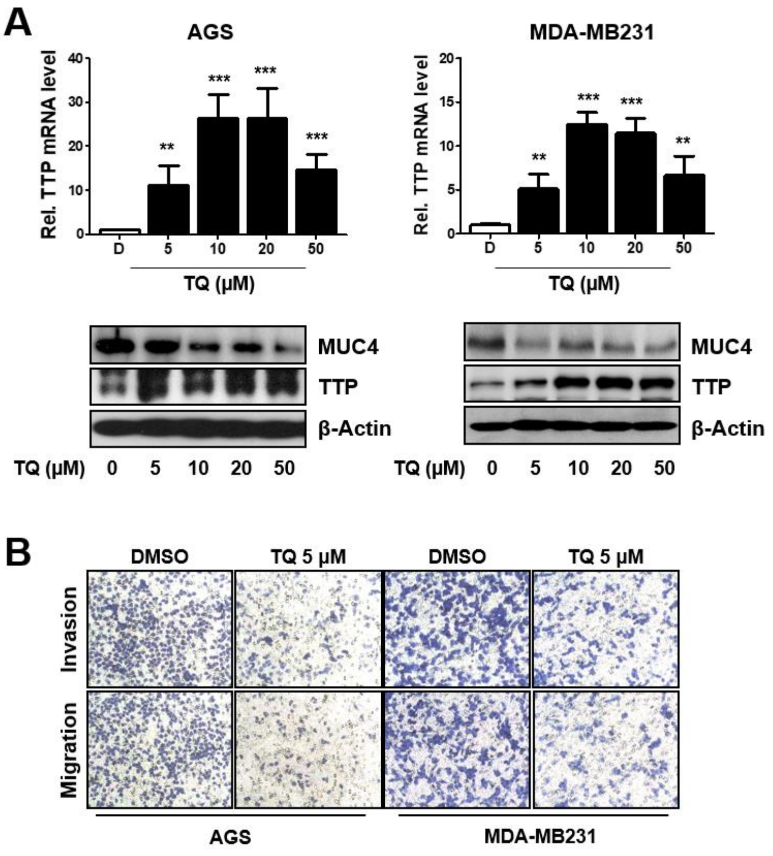

Supplementary Figure S1. (A) AGS and MDA-MB231 cells were treated with TQ in a dose-dependent manner for 4 hours. The expression level of TTP was determined by qRT-PCR and compared with DMSO as a control (upper panel). Protein expression of TTP was determined through the effects of TQ (lower panel). Each bar represents the mean ±S.D. of three independent experiments. (***P*<0.01; ****P*<0.001) (B) AGS and MDA-MB231 cells were treated TQ for 24 hours, invasion and migration assay performed DMSO or TQ 5μM by Boyden chamber assay.

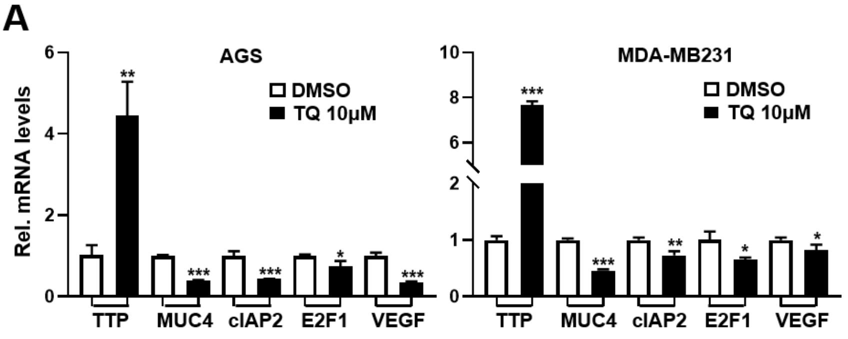

Supplementary Figure S2. (A) AGS (left panel) and MDA-MB231 (right panel) cells treated TQ 10μM for 24 hours, performed TTP, MUC4 and targets(cIAP2, E2F1,VEGF) by qRT-PCR and compared with DMSO as a control. Each bar represents the mean ±S.D. of three independent experiments. (*P<0.05; **P<0.01; ***P<0.001)

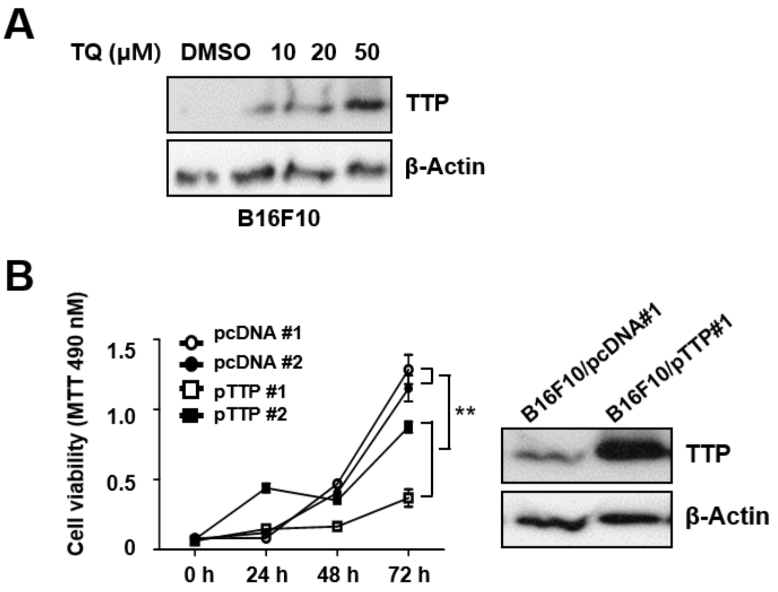

Supplementary Figure S3. Induction of TTP by TQ in mouse B16F10 cells. (A) Protein expression of TTP appeared in a TQ-dose dependent manner in B16F10 cells. (B) TTP overexpression reduce cell viability in B16F10 cells. MTT assay performed in B16F10 cells transfected by TTP overexpression vector. Cells were transfected with pcDNA (control) or TTP (pTTP overexpression vector), and the expression of TTP was examined in these cells. β-actin was detected as a loading control for western blotting. Each bar represents the mean ±S.D. of three independent experiments. (***P*<0.01)

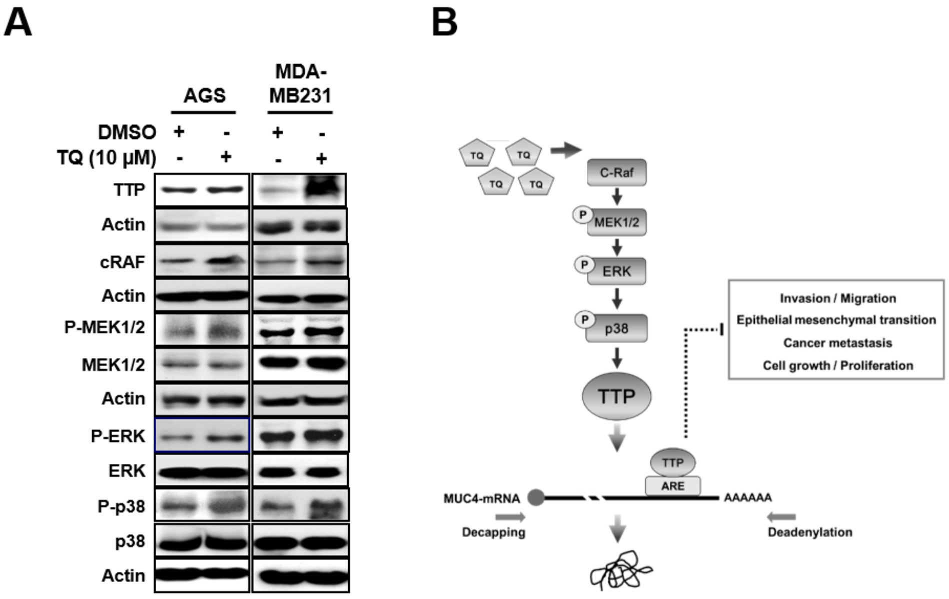

Supplementary Figure S4. (A) AGS and MDA-MB231 cells were treated TQ 10 μM. Western blot assays were used to detect the effects of the Raf-MEK-ERK pathway on TQ-induced TTP expression. (B) A schematic diagram showing a proposed model for a TQ mediated signaling event that induces MUC4 mRNA destabilizing activity of TTP and inhibits tumor progression.

Table S1. qRT-PCR primer sets list.

Primer name	Primer sequence	length
(h) TTP F	5′ CCA AAT ACA AGA CGG AAC TC 3′	20 mer
(h) TTP R	5′ AGG GTG ACA GTG GAA GGT 3′	18 mer
(h) MUC 4 F	5′ GAG GAA TGA CCA GCT GCC TT 3′	20 mer
(h) MUC4 R	5′ AGG GCC AGG GTG TCA TAG AT 3′	20 mer
(h) Actin F	5′CCC TGG AGA AGA GCT ACG AG 3′	20 mer
(h) Actin R	5′ AGG TAG TTT CGT GGA TGC CA 3′	20 mer
(m) TTP F	5′ TCT CTT CAC CAA GGC CAT TC 3′	20 mer
(m) TTP R	5′ GAG AGG AGG TGG TGG GAG TT 3′	20 mer
(m) MUC4 F	5′ CAT ACT AGA GAA CCT GGA CAT G 3′	22 mer
(m) MUC4 R	5′ GAC TTG CTC GAG GGC TGT GCT C 3′	22 mer
(m) Actin F	5′ CTG TCC CTG TAT GCC TCT G 3′	19 mer
(m) Actin R	5′ ATG TCA CGC ACG ATT TCC 3′	18 mer
E-cad F	5′ GCA GTG ACG AAT GTG GTA CC 3′	20 mer
E-cad R	5′ GTG TCT GGC TCC TGG GCA GT 3′	20 mer
N-cad F	5′ GAA TTC AGC ACC CCC CTC AG 3′	20 mer
N-cad R	5′ GCT GCA TAT ATC GAT CTG GG 3′	20 mer
TWIST F	5′ CTA CGC CTT CTC GGT CTG 3′	18 mer
TWIST R	5′ CTT CTC TGG AAA CAA TGA CAT CT 3′	23 mer
SLUG F	5′ TTC ACT CCG AAG CCA AAT G 3′	19 mer
SLUG R	5′ TCT CTC TGT GGG TGT GTG 3′	18 mer
SNAIL F	5′ CCA CAA GCA CCA AGA GTC 3′	18 mer
SNAIL R	5′ TGG CAG TGA GAA GGA TGT 3′	18 mer
ZEB1 F	5′ TGT GCC AAT TTG TTC CTG TA 3′	20 mer
ZEB1 R	5′ TGA GAT GGG AGT CTG GTA AA 3′	20 mer
ZEB2 F	5′ ATC GTG TAA CAA AGA TGA AGA AA 3′	23 mer
ZEB2 R	5′ TCA CAA ATG TCT CAA GTT CTA AA 3′	23 mer
cIAP2 F	5′ CCC TTT TCT TCC CCA TTC AT 3′	20 mer
cIAP2 R	5′ AAA CCA GCA CGA GCA AGA CT 3′	20 mer
E2F1 F	5′ TGC CCT GAG GAG ACC GTA G 3′	19 mer
E2F1 R	5′ GGT GAC ACT ATG GTG CAG AG 3′	20 mer
VEGF F	5′ CGA AGT GGT GAA GTT CAT GGA TGT 3′	24 mer
VEGF R	5′ TCA CCG CCT CGG CTT GTC 3′	18 mer

h; human, m; mouse.
